# Resolution of Extensive Xanthomas Associated With Severe Hypertriglyceridemia via Modified Therapeutic Plasma Exchange

**DOI:** 10.1210/jcemcr/luae054

**Published:** 2024-04-10

**Authors:** Bandari Aljabri, Wafa Saber, Saud Alzahrani, Ashraf Dada

**Affiliations:** Department of Medicine, King Faisal Specialist Hospital and Research Center, Jeddah 23431, Saudi Arabia; Department of Medicine, King Faisal Specialist Hospital and Research Center, Jeddah 23431, Saudi Arabia; Department of Medicine, King Faisal Specialist Hospital and Research Center, Jeddah 23431, Saudi Arabia; Department of Pathology and Laboratory Medicine, King Faisal Specialist Hospital and Research Center, Jeddah 23431, Saudi Arabia; College of Medicine, Al Faisal University, Riyadh 11533, Saudi Arabia

**Keywords:** hypertriglyceridemia, xanthoma, therapeutic plasma exchange, plasmapheresis, case report

## Abstract

Severe hypertriglyceridemia can be manifested by xanthomas. Therapeutic plasma exchange (TPE) is an invasive medical procedure that has been documented as a viable approach for severe hypertriglyceridemia when cases would be refractory to conventional therapies. TPE is mainly an optional therapeutic modality for cases of severe acute pancreatitis or preventing the recurrence of pancreatitis. Beyond this clinical application, data are scarce on TPE utilization in managing cutaneous lesions associated with hypertriglyceridemia. We present a case of severe hypertriglyceridemia accompanied by extensive xanthomas of various types and a history of recurrent pancreatitis. After conventional therapy failed, a modified plasmapheresis regimen was used and was able to achieve a fast and marked reduction in the patient’s serum triglyceride levels with complete resolution of the extensive cutaneous lesions, providing him a newfound comfort he had not experienced in some time and suggesting the regimen potentially could be considered in the treatment of refractory severe hypertriglyceridemia with debilitating cutaneous complications.

## Introduction

Hypertriglyceridemia is a metabolic disorder marked by elevated triglyceride (TG) levels in the bloodstream (1). While it is often asymptomatic, hypertriglyceridemia can give rise to various serious clinical conditions, such as pancreatitis and cardiovascular diseases (2). Furthermore, another observed complication is xanthomas, which are benign skin lesions characterized by the accumulation of lipid-laden foam cells in the dermis, tendons, and other tissues, primarily observed in individuals with TG levels exceeding 1000 mg/dL (11.3 mmol/L) (3). This threshold is classified as severe according to the criteria established by the Endocrine Society for hypertriglyceridemia (1). The risk of developing these complications is typically mitigated by the timely identification of hypertriglyceridemia and the prompt initiation of appropriate therapeutic measures, which typically involve lifestyle modifications and pharmacological interventions (1). In certain clinical scenarios, sporadic reports have emerged highlighting the application of invasive modalities such as therapeutic plasma exchange (TPE) in managing severe acute pancreatitis and preventing recurrent pancreatitis (4-6). However, data on TPE outside this clinical context is sparse rendering it a venue for further study and exploration. We report a case of severe hypertriglyceridemia complicated with extensive xanthomas successfully treated with a modified regimen of TPE while receiving maximal medical therapy.

## Case Presentation

Our case involves a 58-year-old male patient with a history of type 2 diabetes mellitus, hypertension, and dyslipidemia. His dyslipidemia was in the form of severe hypertriglyceridemia for 30 years which was managed by daily doses of atorvastatin 40 mg, fenofibrate 145 mg, and omega-3 supplements, along with lifestyle modifications such as a strict low-fat diet. No invasive measures, including plasmapheresis, were ever attempted to control his hypertriglyceridemia. He had a medical history of 6 episodes of acute pancreatitis, all managed through standard therapy involving fluids and painkillers. His history of diabetes mellitus began at the age of 27 and was initially managed with metformin and sulfonylurea. However, due to poorly controlled blood sugar levels, he eventually transitioned to insulin therapy. During his first visit at the clinic, he was on glargine 38 units at bedtime, and aspart administered with various doses ranging from 30 to 40 units 3 times before meals. There was no history of ischemic heart disease or hypothyroidism. Family history was negative for hypertriglyceridemia or other lipid disorders. He is a nonsmoker and does not consume alcohol. He used to work as a physician in the obstetrics and gynecology field. Unfortunately, he had to stop operating because of disabling painful skin eruptions.

**Figure 1.**
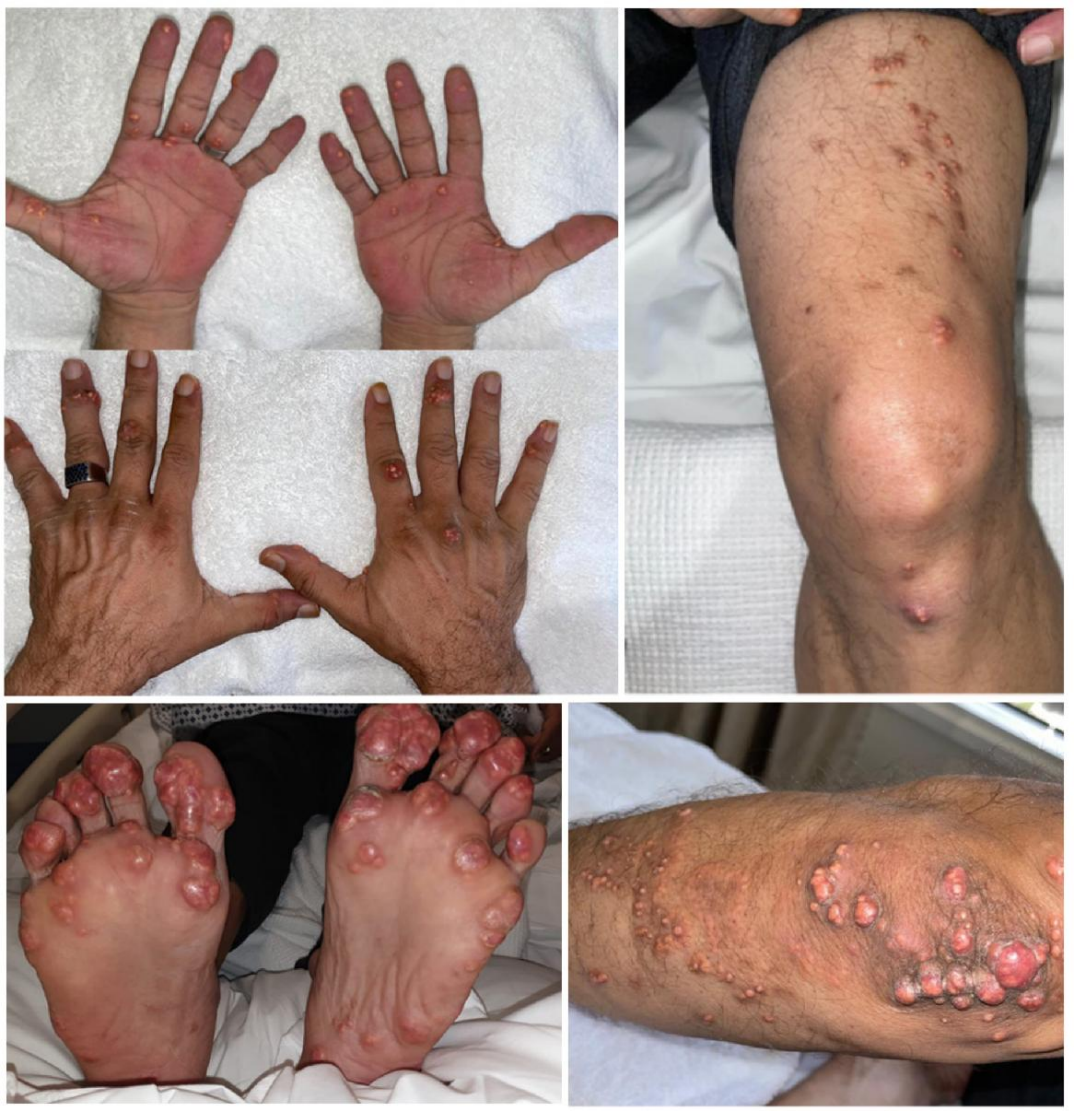
The tuberous xanthomas appreciated at the dorsum and palmar aspect of the hand, tuberous xanthomas mainly noted at the pressure sites of the soles of the feet, along with tubero-eruptive xanthomas at the elbows. Some eruption was found at the knees and thighs.

On physical examination, blood pressure was 145/88, while other vitals were within normal range; his body mass index was 27 kg/m^2^. He had multiple painful tubero-eruptive xanthomas of different sizes. The lesions were extensively distributed to include his elbows, palms, dorsum of the hand, knees with a little extension to the thighs, feet including the soles, and lateral malleolus as well as the entire occipital region (Fig. 1). The pain of the xanthomas was debilitating, to the extent that he could neither operate a vehicle nor get restful sleep, often necessitating assistance to accomplish daily tasks. Throughout the course of his disease, his xanthomas have not shown signs of regression.

## Diagnostic Assessment

Despite being on medical therapy, his lipid profile revealed severely elevated serum TG > 35 mmol/L (> 3000 mg/dL) (normal reference range: < 1.7 mmol/L; 150 mg/dL), total cholesterol 16.9 mmol/L (653.5 mg/dL) (normal reference range: < 5.17 mmol/L; 200 mg/dL), low-density lipoprotein of 1.1 mmol/L (42.5 mg/dL) (target reference range: < 1.8 mmol/L; 70 mg/dL) (7), and high-density lipoprotein of 0.33 mmol/L (12.7 mg/dL) (normal reference range: > 1.55 mmol/L; 60 mg/dL). His glycated hemoglobin (HbA1c) level was 9.5%; alanine aminotransferase was 23 U/L (normal reference range: < 41 U/L), aspartate aminotransferase was 47 U/L (normal reference range: < 40 U/L), and lipase was 107 U/L (normal reference range: 13-60 U/L). Prior laboratory results leading up to the presentation were unavailable. He was admitted for further management of his hypertriglyceridemia.

## Treatment

He was started initially on insulin infusion with no change in his serum TG levels after 3 days. Subsequently, TPE was initiated. After 2 sessions of TPE, his serum TG level dropped from 35 to 11 mmol/L (974.3 mg/dL) with noticeable reduction of pain, improvement of sleep quality, and some reduction in the size of the xanthomas.

**Figure 2.**
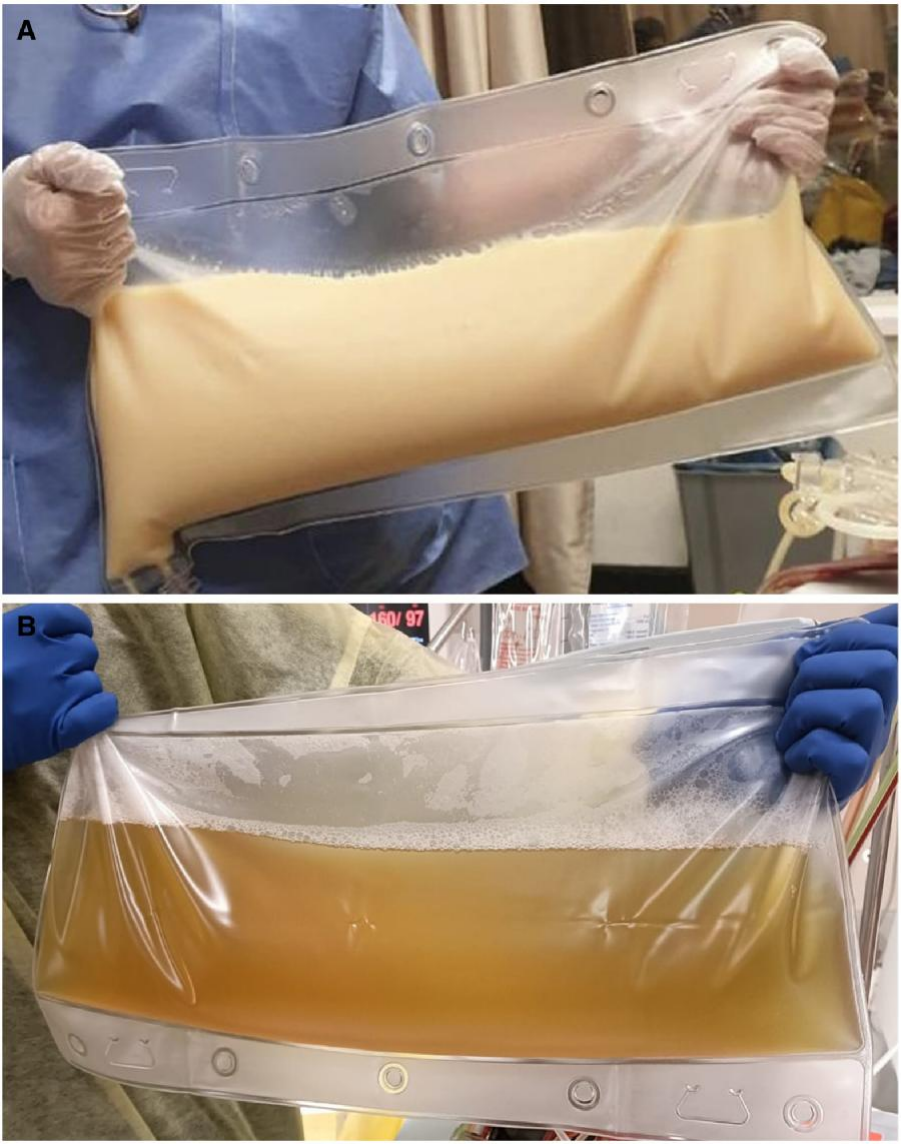
(A) Bag of lipid-filled plasma, removed from our patient during the plasmapheresis, while (B) represents the plasma of a patient with no dyslipidemia.

**Figure 3.**
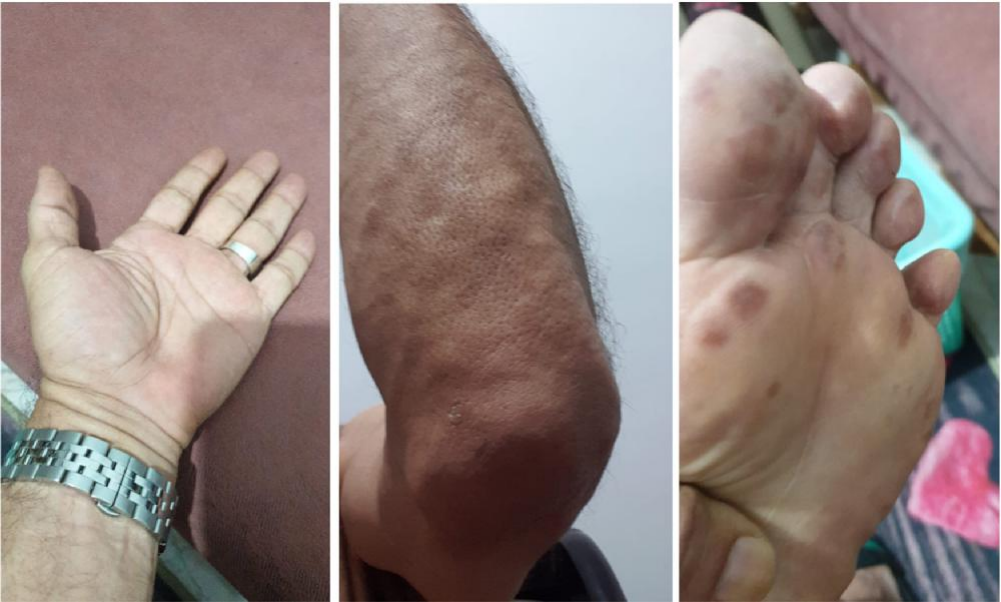
Complete resolution of the previously noted tubero-eruptive xanthomas at the hands, elbows, and feet.

Given the improvement of his clinical condition, several more sessions were arranged to take place every other day. The first 4 sessions posed significant challenges, primarily due to the exceptionally high lipid content in the patient's plasma (Fig. 2), which caused extensive lipid accumulation and clogged multiple apheresis sets. To tackle this issue, the standard TPE protocol was modified by replacing the apheresis set 4 times within a single session. In addition, the session duration was extended from the usual 2 to 7 hours. As a further component of the adapted TPE protocol, a ratio of 1:8 of anticoagulant to whole blood ratio was implemented, utilizing acid citrate dextrose (ACD), in contrast to the standard 1:11 ratio. This adjustment choice was made to minimize the impact on coagulation and accumulation of lipids while simultaneously increasing the administration of calcium gluconate levels to prevent hypocalcemia symptoms. After the fourth session, the patient was able to revert to the standard TPE protocol. This transition became possible due to the substantial reduction in plasma TG levels and continued clinical improvement observed in the patient. At this stage, the patient was able to walk for the first time without difficulty or pain (Fig. 3).

After the sixth session, the frequency of TPE sessions was scheduled to be every 2 weeks with stabilization of his serum TG levels between 7.8 and 16.3 mmol/L (690.8-1443.7 mg/dL), with occasional spikes back to 32 to 35 mmol/L (2834-3011.5 mg/dL). To maintain long-term normalization of the serum TG levels, prevent significant fluctuations, and ensure sustained regression of xanthoma lesions, TPE treatment was extended to a total of 14 sessions.

## Outcome and Follow-Up

After the completion of the fourteenth session, he was followed for 12 months, while he was maintained on a low-fat diet, fenofibrate, and atorvastatin. He had no recurrence of his skin lesions, which were eliminated within 5 months following the initiation of TPE. Additionally, the patient did not develop any new episodes of acute pancreatitis. He did, however, visit the emergency department for abdominal pain, which was attributed to chronic pancreatitis. His serum TG levels remained consistently stable < 3 mmol/L. Laboratory results after 1 year showed TG of 2.7 mmol/L (239.1 mg/dL), total cholesterol of 2.8 mmol/L (108.2 mg/dL), low-density lipoprotein of 0.7 mmol/L (27 mg/dL), and high-density lipoprotein of 0.4 mmol/L (15.4 mg/dL). His HbA1c was 8.2%. Aspartate aminotransferase was 14 U/L and alanine aminotransferase was 9 U/L (Table 1). The exact cause of his lipid disorder could not be explained fully, as it could be a familial genetic disorder in addition to uncontrolled diabetes being a contributing factor; however, genetic testing for those types of disorders is unavailable in our facility.

**Table 1.** Laboratory values before the modified plasma exchange regimen and at a 1-year time point post remission

Laboratory investigation	Before treatment	12 months after treatment	Reference range
TG	> 35 mmol/L (3000 mg/dL)	2.7 mmol/L (239.1 mg/dL)	< 1.7 mmol/L (< 150 mg/dL)
HbA1c	9.1%	8.2%	—
ALT	23 U/L	9 U/L	< 41 U/L
AST	47 U/L	14 U/L	< 40 U/L

Values in parenthesis represent conventional units.

Abbreviations: ALT, alanine aminotransferase; AST, aspartate aminotransferase; HbA1c, glycated hemoglobin; TG, triglyceride;

## Discussion

Xanthomas are a rare complication of severe hypertriglyceridemia, which is defined as TG levels more than 1000 mg/dL (11.3 mmol/L) (1). Xanthomas are characterized by skin manifestations resulting from the pathophysiological accumulation of insoluble lipids within tissues, involving collagen deposition, and the presence of lipid-laden macrophages, giant cells, and inflammatory cells (5, 6). They are typically considered a benign condition, with the other more serious conditions linked to hypertriglyceridemia being acute pancreatitis and atherosclerotic cardiovascular disease (2). Hypertriglyceridemia, a subtype of dyslipidemia, is influenced by, but not limited to, factors such as excess weight, physical inactivity, excessive alcohol intake, the presence of metabolic syndrome or type 2 diabetes mellitus, and specific genetic disorders like familial combined hyperlipidemia, familial hypertriglyceridemia, and familial dysbetalipoproteinemia. Severe hypertriglyceridemia has been suggested to occur more frequently due to polygenic genetic susceptibility in adults rather than being monogenic, compounded by significant exposure to secondary factors, which is believed to be the cause in our case. Initially, the treatment of xanthomas focuses on addressing the underlying hypertriglyceridemia through lifestyle adjustments, smoking cessation, alcohol reduction, dietary modifications, and addressing contributing factors. Implementing effective lifestyle changes can substantially reduce TG levels and subsequently improve the xanthomas. If lifestyle modifications are insufficient, pharmacological interventions are contemplated, and fibrates are commonly chosen as the initial treatment due to their effectiveness in reducing TG levels by up to 50%; fenofibrate is the preferred choice in this case as gemfibrozil should not be combined with statin medications due to the risk of myopathy. Other effective medications include statin medications, niacin, bile acid sequestrants, and omega-3 fatty acids (1, 2, 8, 9). By implementing lifestyle modifications and utilizing pharmacological interventions, eruptive xanthomas can exhibit a positive response within a few weeks. On the other hand, tuberous xanthomas may require several months for noticeable improvement, and tendinous xanthomas may take years to resolve or, rarely, persist indefinitely (10). Our case illustrates a considerable challenge, as hypertriglyceridemia remained severe and subsequently xanthomas persisted despite maximal medical treatment. The patient had already been initiated on a combination of statins, fenofibrate, and omega-3 treatments, in addition to lifestyle adjustments, for an extended period, with no significant reduction in TG levels or improvement in xanthomas. Additional extensive treatment measures encompassed the utilization of intravenous insulin infusion, a well-documented and known approach. However, we continued insulin infusion for 72 hours, achieving a target blood sugar range of 10 to 12 mmol/L. Despite this, there was no change in the TG levels (2, 11). Other approaches have been reported in the medical literature supporting the use of surgical interventions for severe xanthomas cases (12, 13). However, in our specific case, TPE was still an option, and surgical intervention would carry a significant risk because of the extensive distribution of xanthomas. Moreover, the efficacy of surgical removal of xanthomas may be uncertain and provide only temporary relief. This is because surgical interventions do not address the underlying hypertriglyceridemia, making recurrence likely. Similar reservations apply to other alternative invasive therapies described in the literature, such as cryotherapy (14).

Considering these limitations, we explored TPE as a strategy for addressing the refractory hypertriglyceridemia and the associated xanthomas, aiming for a comprehensive and lasting solution. TPE effectiveness in reducing chylomicron and TG levels is supported by studies involving acute and recurrent pancreatitis cases where hypertriglyceridemia was the underlying cause (15, 16). Nonetheless, while TPE has proven effective for managing hypertriglyceridemia-induced pancreatitis, its application in other manifestations associated with severe hyperglyceridemia, particularly xanthomas, remains largely underexplored in scientific literature. Due to the extensive nature of the patient’s hypertriglyceridemia, employing TPE for management was a challenge. However, through the implementation of the aforementioned modifications to the conventional TPE protocol, the intricate technical challenges encountered during the TPE procedure were addressed. As a result, TG levels improved and the xanthomas have also resolved. To solidify these results, further clinical investigation on the expanded use of modified TPE in severe hypertriglyceridemia is essential.

## Learning Points

- TPE is a valuable tool in treating and managing severe hypertriglyceridemia-related skin manifestations, providing an important method to control hypertriglyceridemia despite its cost and limited availability.
- Overcoming technical challenges in plasmapheresis for severe hypertriglyceridemia involves adapting the treatment approach by lengthening sessions, making frequent exchanges of the pheresis bags, changing the anticoagulant to whole blood ratio to improve lipid clearance, and taking precautions to prevent hypocalcemia by increasing the administration of calcium gluconate.
- A multidisciplinary approach is crucial for optimal plasmapheresis management in xanthomas. It involves collaborative decision-making for treatment initiation, session planning, and monitoring for potential complications.

## Contributors

All authors made individual contributions to authorship. S.A. was involved in the diagnosis and management of the patient. B.A. and W.S. were involved in preparing and submitting the manuscript and its accompanying images. A.D. was involved in the management of the patient, providing expert input in the plasmapheresis segment, and reviewing the manuscript. All authors reviewed and approved the final draft.

## Data Availability

Data sharing is not applicable to this article as no datasets were generated or analyzed during the current study.

## Abbreviations

HbA1c: glycated hemoglobin
TG: triglycerides
TPE: therapeutic plasma exchange
